# Development and validation of risk prediction equations to estimate survival in patients with colorectal cancer: cohort study

**DOI:** 10.1136/bmj.j2497

**Published:** 2017-06-15

**Authors:** Julia Hippisley-Cox, Carol Coupland

**Affiliations:** 1 Division of Primary Care, University Park, Nottingham NG2 7RD, UK

## Abstract

**Objective** To develop and externally validate risk prediction equations to estimate absolute and conditional survival in patients with colorectal cancer.

**Design** Cohort study.

**Setting** General practices in England providing data for the QResearch database linked to the national cancer registry.

**Participants** 44 145 patients aged 15-99 with colorectal cancer from 947 practices to derive the equations. The equations were validated in 15 214 patients with colorectal cancer from 305 different QResearch practices and 437 821 patients with colorectal cancer from the national cancer registry.

**Main outcome measures** The primary outcome was all cause mortality and secondary outcome was colorectal cancer mortality.

**Methods** Cause specific hazards models were used to predict risks of colorectal cancer mortality and other cause mortality accounting for competing risks, and these risk estimates were combined to obtain risks of all cause mortality. Separate equations were derived for men and women. Several variables were tested: age, ethnicity, deprivation score, cancer stage, cancer grade, surgery, chemotherapy, radiotherapy, smoking status, alcohol consumption, body mass index, family history of bowel cancer, anaemia, liver function test result, comorbidities, use of statins, use of aspirin, clinical values for anaemia, and platelet count. Measures of calibration and discrimination were determined in both validation cohorts at 1, 5, and 10 years.

**Results** The final models included the following variables in men and women: age, deprivation score, cancer stage, cancer grade, smoking status, colorectal surgery, chemotherapy, family history of bowel cancer, raised platelet count, abnormal liver function, cardiovascular disease, diabetes, chronic renal disease, chronic obstructive pulmonary disease, prescribed aspirin at diagnosis, and prescribed statins at diagnosis. Improved survival in women was associated with younger age, earlier stage of cancer, well or moderately differentiated cancer grade, colorectal cancer surgery (adjusted hazard ratio 0.50), family history of bowel cancer (0.62), and prescriptions for statins (0.77) and aspirin (0.83) at diagnosis, with comparable results for men. The risk equations were well calibrated, with predicted risks closely matching observed risks. Discrimination was good in men and women in both validation cohorts. For example, the five year survival equations on the QResearch validation cohort explained 45.3% of the variation in time to colorectal cancer death for women, the D statistic was 1.86, and Harrell’s C statistic was 0.80 (both measures of discrimination, indicating that the scores are able to distinguish between people with different levels of risk). The corresponding results for all cause mortality were 42.6%, 1.77, and 0.79.

**Conclusions** Risk prediction equations were developed and validated to estimate overall and conditional survival of patients with colorectal cancer accounting for an individual’s clinical and demographic characteristics. These equations can provide more individualised accurate information for patients with colorectal cancer to inform decision making and follow-up.

## Introduction

Traditional estimates of cancer survival provide important information for guidelines, planning treatment, follow-up, and ongoing surveillance for different types of cancer. Relative survival estimates are traditionally used to cancel out changes in competing causes of death so that changes in prevention and treatment strategies can be compared over time and between populations.^1^ The relative net survival essentially removes the competing cause of death, comparing survival in patients with cancer with the expected survival in people without cancer. Relative survival estimates are usually based on analyses of cancer registry data alone and presented as a series of tables taking account of one or two factors (eg, the patient’s age and sex, or the stage of cancer at diagnosis).^2^ While such estimates are useful for researchers and policy makers, they are less relevant for patients and clinicians, who tend to be interested in individualised absolute survival to support treatment decisions and make informed and meaningful choices.

Several prognostic models exist, which predict overall mortality or survival for colorectal cancer.^3^ ^4^ ^5^ ^6^ Although these models incorporate additional clinical variables and show improved performance compared with the Tumour Nodes Metastases (TNM) system, they tend to be limited to specific patient groups (such as those with stage 3 disease^5^ or those undergoing curative intent surgery^6^), be based on selected populations of patients recruited to clinical trials^5^ ^6^ or attending specialist centres, and have relatively poor discrimination^5^ ^6^ or are not published.^4^ None of these models can be applied to all patients with colorectal cancer, incorporate family history or specific comorbidities, predict survival over periods longer than five years, or update predictions based on the number of years survived since diagnosis.^4^ ^5^ ^6^

Realistic estimates of overall survival are particularly important for patients with a diagnosis of cancer who need to make decisions about the risks and benefits of active versus supportive treatment (eg, whether to have surgery, chemotherapy, radiotherapy, or palliative care).^7^ Current evidence suggests that issues around survival are only discussed properly in approximately 30% of cases.^8^ This may reflect a lack of robust information on survival, which takes account of patient characteristics and the likely effect of different treatments.^9^

Therefore, we decided to develop and externally validate a set of prediction equations to quantify absolute survival for patients with colorectal cancer. We accounted for other clinical factors available through routine linkage of cancer registry data to primary care electronic health records. We decided to include estimates of conditional survival since this provides a more relevant measure of survival among those surviving the first year, especially when the initial prognosis is poor (ie, advanced stage colorectal cancer).^10^ Such estimates can be used to provide better information for patients and clinicians to help inform treatment and other life decisions.^1^

## Methods

### Study design and data source

We undertook a cohort study to derive and validate the risk equations in a large population of primary care patients with colorectal cancer using the UK QResearch database (version 41, www.qresearch.org). A second external validation was undertaken using a separate cohort of patients included on the national cancer registry. QResearch is a continually updated patient level pseudonymised database, with data extending back to 1989. It includes data from over 1200 general practices covering a population of more than 22 million patients, collected during the course of routine healthcare. The data includes demographic information, smoking status, alcohol consumption, diagnoses, prescriptions, referrals, laboratory test results, and clinical values. QResearch has been used for a wide range of clinical research, such as the development and validation of risk prediction models,^11^ including risk of existing but undiagnosed cancer^12^ and future cancers.^13^

QResearch is linked at individual patient level to national cancer registry data supplied by Public Health England (PHE) and the mortality register supplied by the Office for National Statistics (ONS).^1^ The mortality register includes information on the date of death as well as the primary and underlying cause of death. The PHE cancer registry currently includes all cancers registered in England between 1990 and 2014, with follow-up for mortality until 31 December 2015. The PHE cancer registry includes information on year of birth, age at diagnosis, date of death, sex, ethnicity, Townsend deprivation fifth, date of cancer diagnosis, binary variables for cancer treatments such as surgery and chemotherapy undertaken within a year of diagnosis, tumour location, tumour growth behaviour, cancer grade, cancer stage, and whether the cancer diagnosis was only present on a death certificate. The PHE cancer registry, however, does not include the cause of death or other variables such as smoking status, alcohol consumption, body mass index, comorbidities, or prescribed drugs.

### Cohort selection

We included all QResearch practices using the Egton Medical Information Systems (EMIS) for at least one year during the study period. We randomly allocated three quarters of these practices to the derivation dataset and the remaining quarter to a validation dataset. In both datasets we identified open cohorts of patients registered with eligible practices between 1 January 1998 and 31 December 2014.

### Inclusion and exclusion criteria

We included patients registered with QResearch with a first recorded diagnosis of colorectal cancer on the linked cancer registry data between 1 January 1998 and 31 December 2014. To identify cases of colorectal cancer we used ICD-10 (international classification of diseases, 10th revision) codes (C18, C19, and C20).^14^ ^15^ ^16^ The analysis was restricted to patients aged 15 to 99 years at diagnosis who had a first diagnosis of colorectal cancer during the period of registration with the practice, ensuring that each patient was registered with the practice for at least one year before cancer diagnosis. We used the date of cancer diagnosis from the linked cancer registry data as the index date for entry to the cohort, and patients were followed up until the earliest of the date of death or 31 December 2015 to ensure a minimum of 12 months’ follow-up after diagnosis.

We excluded patients where the growth behaviour for the index cancer diagnosis was coded as benign and those where the diagnosis was made on or after death, because the duration of survival is unknown.^14^ ^15^

### External validation cohort

For the separate external validation cohort, we identified patients aged 15 to 99 years with a diagnosis of colorectal cancer recorded on the PHE cancer registry who were not registered with QResearch practices at the time of diagnosis, excluding those where the growth behaviour of their cancer was coded as benign and those with a death certificate only diagnosis.

### Outcomes

Our primary outcome was all cause mortality. Our secondary outcome was colorectal cancer mortality. We used the primary and underlying causes of death on the linked ONS record to identify deaths from colorectal cancer.

### Predictor variables

Box 1 lists the predictor variables we examined, based on established factors affecting mortality after a diagnosis of cancer and those affecting all cause mortality. We did this so that the absolute risk estimates will be able to reflect these factors.

**Box 1: Predictor variables**

**Variables derived from Public Health England cancer registry**

Age at diagnosis^15^

Self assigned ethnicity (white or not recorded, Indian, Pakistani, Bangladeshi, other Asian, Caribbean, black African, Chinese, or other)

Townsend deprivation score

Cancer stage^15^ ^16^ classified using Tumour Nodes Metastases classification (version 7)^17^ (stage 1: local involvement only, stage 2: extension to adjacent tissue, stage 3: lymph node involvement, stage 4: metastasis, or not recorded)

Cancer grade (well differentiated, moderately differentiated, poorly differentiated, and undifferentiated, or not recorded)

Colorectal cancer surgery within a year of diagnosis

Chemotherapy within a year of diagnosis

Radiotherapy within a year of diagnosis

**Variables derived from patients linked GP record**

Most recent smoking status before diagnosis (non-smoker, former smoker, light smoker <10/day, moderate smoker 10-19/day, or heavy smoker ≥20/day)

Most recent alcohol status before diagnosis (non-drinker, trivial: <1 unit/day, light: 1-2 units/day, or moderate or heavy: >3 units/day)

Family history of bowel cancer (yes or no)

Previous cancer (other than colorectal) (yes or no)

Cardiovascular disease (yes or no)

Diabetes (type 1, type 2, or no diabetes)

Chronic renal disease (yes or no)

Chronic liver disease (yes or no)

Inflammatory bowel disease (yes or no)

Chronic obstructive pulmonary disease (yes or no)

Venous thromboembolism (yes or no)

Statin use^18^ ^19^ (yes or no)

Aspirin use^20^ ^21^ (yes or no)

Abnormal platelet count defined as values >480×10^9^/L

Abnormal liver function test result, defined as either γ glutamyltransferase or alanine aminotransferase or bilirubin more than three times normal^22^ based on the value closest to cancer diagnosis (yes or no)

Anaemia, defined as haemoglobin <110 g/L (yes or no)

Most recent body mass index before diagnosis

Self assigned ethnicity where not recorded on the PHE cancer registry (white or not recorded, Indian, Pakistani, Bangladeshi, other Asian, Caribbean, black African, Chinese, or other)

For body mass index, smoking status, and alcohol consumption, we used the most recent value before cancer diagnosis. For blood tests, we used the values recorded closest to the date of diagnosis, selecting from those recorded within 12 months either side of the diagnosis date. For comorbidities, family history, use of statins, and use of aspirin we used values recorded before diagnosis. We also included the Townsend deprivation score, which is an area level score based on the patients’ postcode.^23^ Originally developed by Townsend,^23^ it includes unemployment (as a percentage of those aged 16 or more who are economically active), non-car ownership (as a percentage of all households), non-home ownership (as a percentage of all households), and household overcrowding. These variables are measured for a given area of approximately 120 households, using the 2011 census, and combined to give a Townsend score for that area. A greater Townsend score implies a greater level of deprivation.

### Derivation of the predictive models

We developed the risk models using cause specific hazard models to account for competing risks such as death from other causes. Firstly, we used multiple imputation with chained equations to replace missing values for continuous values (body mass index) and categorical variables (smoking status, alcohol consumption, cancer stage, and cancer grade).^24^ ^25^ ^26^ We carried out five imputations and included all potential predictor variables in each imputation model along with the outcome variables, the Nelson-Aalen estimator of the baseline cumulative hazard,^27^ and the interaction terms.

Secondly, we fitted two separate cause specific Cox models: one for deaths from colorectal cancer and one for deaths from other causes. To allow for clustering of patients within general practices, we used robust variance estimates. We used fractional polynomial terms^28^ to model non-linear risk relations for age and body mass index. We used Rubin’s rules to combine the regression coefficients across the five imputed datasets.^29^ Variables were retained in the prediction models if they had a hazard ratio of <0.85 or >1.15 (for binary variables) and were statistically significant at the 0.01 level. We tested for two way interactions between age and each predictor; cancer stage and each predictor; and combinations of cancer grade, surgery, and chemotherapy. We included significant interactions (where P<0.01). A formula^30^ was used to derive the cumulative incidence function for colorectal cancer mortality accounting for competing risk of death from other causes using estimates obtained from the two Cox models. This formula multiplies the hazard contribution for colorectal cancer mortality at a given time by the probability of death from other causes at that time and then sums these values across the time period of interest. We used the same method to derive the cumulative incidence function for deaths from other causes, accounting for competing risk of death from colorectal cancer. We added these two cumulative incidence functions to give estimates of the risk of death from all causes. We obtained values of these estimates at 1, 5, and 10 years after diagnosis and hence derived estimates of absolute survival probabilities at 1, 5, and 10 years after diagnosis.

### Conditional survival

#### Overall survival

We calculated overall survival estimates conditional on having survived a given number of years after diagnosis (X) for patients who had already survived Y years since diagnosis by dividing the absolute survival at X years by the absolute overall survival estimates at Y years.^31^

#### Cause specific mortality

To calculate cause specific mortality estimates at a given number of years after diagnosis (X) for patients who had already survived Y years since diagnosis, we calculated the cumulative risk at X years minus the cumulative risk at Y years and divided this difference by the overall survival at Y years (ie, ((cumulative risk at X years)–(cumulative risk at Y years))/overall survival at Y years). In this way it is possible to calculate predictions for overall survival as well as cause specific mortality conditional on survival for any number of years for each year until 10 years.

### Landmark analysis

Patients were recorded on the PHE cancer registry as having received chemotherapy or colorectal surgery if this was undertaken within a year of the date of diagnosis of cancer but the precise date of treatment was unavailable. As a further analysis we therefore undertook a landmark analysis^32^ to avoid immortal time bias (which would tend to overestimate the benefit of treatment)^33^ and compared hazard ratios for cancer treatments with the main analysis. We assigned a landmark date that was 365 days after cancer diagnosis. Deaths that occurred between the diagnosis date and landmark date were excluded from these analyses.

### Validation of the predictive models

We used multiple imputation in the QResearch validation cohort to replace missing values using the same imputation model as in the derivation cohort. In the PHE validation cohort, we used multiple imputation to replace missing values for cancer stage and grade only. The risk equations were applied to both the QResearch and the PHE validation cohort. We assumed zero values for variables in the model that were not recorded on the PHE dataset (these variables were family history of bowel cancer, raised platelet count, abnormally raised liver function test result, statin use, aspirin use, cardiovascular disease, diabetes, renal disease, and chronic obstructive pulmonary disease). A value of 25 kg/m^2^ was assumed for body mass index.

We calculated R^2^ values (explained variation in time to death^34^), D statistics^35^ (measure of discrimination, where higher values indicate better discrimination), and Harrell’s C statistics over 1, 5, and 10 years. Harrell’s C statistic^36^ is a measure of discrimination that is similar to the receiver operating characteristic statistic but takes account of the censored nature of the data. Using Rubin’s rules we combined these model performance measures across imputed datasets. We assessed calibration for all cause mortality by comparing the observed risks at 1, 5, and 10 years with the mean predicted risks by 10th of predicted risk using Kaplan-Meier estimates of observed risk. Calibration of the colorectal mortality estimates was assessed by comparing non-parametric estimates of cumulative incidence, which approximate observed risk accounting for competing risk of death from other causes,^37^ with the mean predicted risks across 10ths of predicted risk. A model is well calibrated if predicted risks closely approximate the observed risks.

### Survival rates

In each of the three cohorts we calculated the age standardised observed survival and the relative net survival for patients from the date of diagnosis for comparisons with other studies.^14^ ^38^ Relative survival is the ratio of the overall survival for a cohort of patients with cancer to the expected survival in the general population matched by age, sex, and calendar year. We used background rates obtained from ONS. We also calculated survival estimates for patients, conditional on the patient surviving for each year after diagnosis.^14^ ^39^

### Decision curve analysis

We used decision curve analysis in the QResearch validation cohort (accounting for competing risks) to evaluate the net benefits of the new risk equations for deaths from colorectal cancer and from other causes.^40^ ^41^ ^42^ This approach assesses the benefits of correctly detecting people who will have an event compared with the harms from a false positive classification (which could lead to unnecessary treatment). The net benefit of a risk equation at a given risk threshold is calculated by calculating the difference between the proportion of true positives and the proportion of false positives multiplied by the odds of the risk threshold. We calculated the net benefits across a range of threshold probabilities and compared these with alternative strategies such as not treating anyone or treating everyone. In general, the strategy with the highest net benefit at any given risk threshold is considered to have the most clinical value.

To maximise power and generalisability we included all the eligible patients in each database. All analyses were done using STATA (version 14.1). We adhered to the transparent reporting of a multivariable prediction model for individual prognosis or diagnosis (TRIPOD) statement for reporting.^43^ Further details can be found in the study protocol.^44^

### Patient involvement

Patients were not involved in setting the research question. A patient representative reviewed the study protocol and advised on the design and outcome measures and the lay summary. Patient representatives from the QResearch advisory board have written the information for patients on the QResearch website about the use of the database for research in general. They have also advised on dissemination, including the use of lay summaries describing the research and its results.

## Results

### Overall study population

Overall, 1252 practices in England participating in QResearch met our practice inclusion criteria, of which 947 were randomly assigned to the derivation dataset with the remaining 305 practices assigned to the validation cohort. Overall we identified 525 416 patients with a diagnosis of colorectal cancer on the Public Health England (PHE) cancer registry between 1998 and 2014. We excluded 475 patients who were aged less than 15 years or more than 99 years, two patients where the behaviour of the tumour was benign, and 18 248 patients where the diagnosis was made on or after death. Of the remaining 497 180 patients, 44 145 were registered with QResearch practices in the derivation cohort and 15 214 were in the QResearch validation cohort, leaving 437 821 patients for inclusion in the PHE validation cohort.

### Baseline characteristics

Table 1 shows the baseline characteristics of men and women with colorectal cancer for each cohort using data from the cancer registry. Table 2 shows additional data available from the general practice record. For the 44 145 patients in the derivation cohort, the mean age at diagnosis was 71.6 years. In total, 28 220 (63.9%) had colon cancer, 12 674 (28.7%) had rectal cancer, and 3251 (7.4%) had rectosigmoid cancer; 32 237 (73.0%) had cancer stage recorded and 34 370 (77.9%) had grade recorded; 33 268 (75.4%) were treated with colorectal surgery and 13 567 (30.7%) had chemotherapy. Of the 13 567 patients who had chemotherapy, 327 (2.4%) had stage 1 cancer, 2235 (16.5%) had stage 2 cancer, 5717 (42.1%) had stage 3 cancer, and 2277 (20.4%) had stage 4 cancer, and in 2516 (18.5%) stage was not recorded. Smoking status was recorded in 41 449 (93.9%) of patients, ethnicity in 38 544 (87.3%), and body mass index in 36 979 (83.8%). In the derivation cohort, 17 642 (40.0%) had complete data for all the variables compared with 5716 (37.6%) in the validation cohort. Table 1 in the web appendix shows summary statistics of predictor variables for patients with complete data compared with those with one or more missing values.

**Table 1 tbl1:** Baseline characteristics of men and women with colorectal cancer aged 15-99 years in the QResearch derivation cohort and QResearch and Public Health England (PHE) validation cohorts based on information recorded in the cancer registry. Values are numbers (percentages) unless stated otherwise

Characteristics	Women				Men		
	QResearch derivation cohort	QResearch validation cohort	PHE validation cohort		QResearch derivation cohort	QResearch validation cohort	PHE validation cohort
Total No of patients	19 708	6800	196 239		24 437	8414	241 582
Mean (SD) age at diagnosis (years)	72.5 (12.8)	72.7 (12.8)	72.5 (12.9)		70.9 (11.5)	70.9 (11.6)	70.5 (11.7)
Age group (years):							
15-19	17 (0.1)	4 (0.1)	181 (0.1)		6 (0.0)	3 (0.0)	119 (0.0)
20-29	67 (0.3)	33 (0.5)	817 (0.4)		65 (0.3)	16 (0.2)	775 (0.3)
30-39	241 (1.2)	68 (1.0)	2201 (1.1)		215 (0.9)	93 (1.1)	2420 (1.0)
40-49	729 (3.7)	251 (3.7)	7279 (3.7)		807 (3.3)	266 (3.2)	8113 (3.4)
50-59	1960 (9.9)	650 (9.6)	20 146 (10.3)		2679 (11.0)	931 (11.1)	27 545 (11.4)
60-69	4097 (20.8)	1408 (20.7)	40 588 (20.7)		6376 (26.1)	2197 (26.1)	64 424 (26.7)
70-79	6069 (30.8)	2084 (30.6)	59 928 (30.5)		8356 (34.2)	2854 (33.9)	82 423 (34.1)
80-89	5371 (27.3)	1896 (27.9)	53 492 (27.3)		5257 (21.5)	1817 (21.6)	49 535 (20.5)
90-99	1157 (5.9)	406 (6.0)	11 607 (5.9)		676 (2.8)	237 (2.8)	6228 (2.6)
Ethnicity:							
Recorded	17005 (86.3)	5896 (86.7)	144409 (73.6)		21539 (88.1)	7435 (88.4)	184127 (76.2)
White or not recorded	19150 (97.2)	6603 (97.1)	191 802 (97.7)		23 699 (97.0)	8183 (97.3)	235 692 (97.6)
Indian	84 (0.4)	28 (0.4)	756 (0.4)		147 (0.6)	40 (0.5)	1184 (0.5)
Pakistani	32 (0.2)	14 (0.2)	373 (0.2)		57 (0.2)	10 (0.1)	495 (0.2)
Bangladeshi	40 (0.2)	12 (0.2)	139 (0.1)		48 (0.2)	14 (0.2)	166 (0.1)
Other Asian	53 (0.3)	14 (0.2)	297 (0.2)		60 (0.2)	18 (0.2)	404 (0.2)
Black Caribbean	123 (0.6)	54 (0.8)	900 (0.5)		168 (0.7)	61 (0.7)	1097 (0.5)
Black African	49 (0.2)	18 (0.3)	386 (0.2)		67 (0.3)	22 (0.3)	429 (0.2)
Chinese	27 (0.1)	13 (0.2)	302 (0.2)		34 (0.1)	17 (0.2)	377 (0.2)
Other	150 (0.8)	44 (0.6)	1284 (0.7)		157 (0.6)	49 (0.6)	1738 (0.7)
Year of diagnosis:							
1998-2005	7462 (37.9)	2625 (38.6)	87 301 (44.5)		8786 (36.0)	3174 (37.7)	104 651 (43.3)
2006-14	12 246 (62.1)	4175 (61.4)	108 938 (55.5)		15 651 (64.0)	5240 (62.3)	136 931 (56.7)
Type of cancer:							
Colon	13 641 (69.2)	4580 (67.4)	134 832 (68.7)		14 579 (59.7)	5078 (60.4)	143 254 (59.3)
Rectal	4735 (24.0)	1710 (25.1)	47 966 (24.4)		7939 (32.5)	2694 (32.0)	78 821 (32.6)
Rectosigmoid	1332 (6.8)	510 (7.5)	13 441 (6.8)		1919 (7.9)	642 (7.6)	19 507 (8.1)
Townsend deprivation fifth:							
1 (most affluent)	4505 (22.9)	1594 (23.4)	37 911 (19.3)		5649 (23.1)	2076 (24.7)	48 605 (20.1)
2	4612 (23.4)	1460 (21.5)	42 934 (21.9)		5775 (23.6)	1788 (21.3)	52 517 (21.7)
3	3968 (20.1)	1419 (20.9)	43 032 (21.9)		4837 (19.8)	1636 (19.4)	51 619 (21.4)
4	3559 (18.1)	1275 (18.8)	39 510 (20.1)		4236 (17.3)	1484 (17.6)	47 309 (19.6)
5 (most deprived)	3064 (15.5)	1052 (15.5)	32 852 (16.7)		3940 (16.1)	1430 (17.0)	41 532 (17.2)
Cancer stage at diagnosis:							
Stage recorded	14 193 (72.0)	4724 (69.5)	140 613 (71.7)		18 044 (73.8)	5918 (70.3)	177 401 (73.4)
1	2030 (10.3)	697 (10.3)	19 542 (10.0)		2763 (11.3)	938 (11.1)	26 977 (11.2)
2	4926 (25.0)	1592 (23.4)	47 791 (24.4)		5783 (23.7)	1942 (23.1)	57 679 (23.9)
3	4715 (23.9)	1645 (24.2)	48 194 (24.6)		6205 (25.4)	2052 (24.4)	60 040 (24.9)
4	2522 (12.8)	790 (11.6)	25 086 (12.8)		3293 (13.5)	986 (11.7)	32 705 (13.5)
Cancer grade at diagnosis:							
Grade recorded	15 077 (76.5)	5066 (74.5)	147 796 (75.3)		19 293 (78.9)	6508 (77.3)	189 146 (78.3)
Well differentiated	1110 (5.6)	366 (5.4)	10 499 (5.4)		1306 (5.3)	479 (5.7)	13 168 (5.5)
Moderately differentiated	11 162 (56.6)	3787 (55.7)	109 278 (55.7)		15 003 (61.4)	5122 (60.9)	147 237 (60.9)
Poorly differentiated	2777 (14.1)	900 (13.2)	27 589 (14.1)		2949 (12.1)	893 (10.6)	28 332 (11.7)
Undifferentiated	28 (0.1)	13 (0.2)	430 (0.2)		35 (0.1)	14 (0.2)	409 (0.2)
Treatment within 12 months of diagnosis:							
Surgery	14 729 (74.7)	4971 (73.1)	145 376 (74.1)		18 539 (75.9)	6276 (74.6)	182 573 (75.6)
Chemotherapy	5504 (27.9)	1849 (27.2)	52 228 (26.6)		8063 (33.0)	2665 (31.7)	76 495 (31.7)
Radiotherapy	2325 (11.8)	870 (12.8)	22 255 (11.3)		4116 (16.8)	1361 (16.2)	39 175 (16.2)

**Table 2 tbl2:** Baseline characteristics of patients with colorectal cancer aged 15-99 years in QResearch derivation and validation cohorts using information derived from the linked primary care data. Values are numbers (percentages)

Characteristics	Women			Men	
	Derivation cohort	Validation cohort		Derivation cohort	Validation cohort
Total No of patients	19 708	6800		24 437	8414
Smoking status:					
Recorded	18 405 (93.4)	6320 (92.9)		23 044 (94.3)	7936 (94.3)
Non-smoker	11 416 (57.9)	3901 (57.4)		10 115 (41.4)	3473 (41.3)
Former smoker	4898 (24.9)	1691 (24.9)		9806 (40.1)	3347 (39.8)
Light smoker	1195 (6.1)	433 (6.4)		1994 (8.2)	693 (8.2)
Moderate smoker	584 (3.0)	191 (2.8)		620 (2.5)	214 (2.5)
Heavy smoker	312 (1.6)	104 (1.5)		509 (2.1)	209 (2.5)
Alcohol consumption:					
Recorded	16 398 (83.2)	5590 (82.2)		20 776 (85.0)	7102 (84.4)
Non-drinker	7460 (37.9)	2466 (36.3)		5160 (21.1)	1779 (21.1)
Trivial, <1 unit/day	5701 (28.9)	2015 (29.6)		6025 (24.7)	2079 (24.7)
Light, 1-2 units/day	1873 (9.5)	645 (9.5)		3584 (14.7)	1225 (14.6)
Moderate or heavy, >3 units/day	1355 (6.9)	462 (6.8)		5965 (24.4)	2010 (23.9)
Medical history:					
Family history of bowel cancer	514 (2.6)	186 (2.7)		481 (2.0)	159 (1.9)
Other cancer	1570 (8.0)	553 (8.1)		1912 (7.8)	632 (7.5)
CVD	3054 (15.5)	1067 (15.7)		5701 (23.3)	1984 (23.6)
Type 1 diabetes	29 (0.1)	15 (0.2)		68 (0.3)	25 (0.3)
Type 2 diabetes	2207 (11.2)	766 (11.3)		3617 (14.8)	1236 (14.7)
Chronic renal disease	266 (1.3)	80 (1.2)		343 (1.4)	122 (1.4)
Chronic liver disease	143 (0.7)	55 (0.8)		242 (1.0)	79 (0.9)
Inflammatory bowel disease	301 (1.5)	96 (1.4)		366 (1.5)	122 (1.4)
COPD	1035 (5.3)	391 (5.8)		1930 (7.9)	680 (8.1)
VTE	901 (4.6)	295 (4.3)		939 (3.8)	357 (4.2)
Prescribed statins at diagnosis	4258 (21.6)	1412 (20.8)		7246 (29.7)	2440 (29.0)
Prescribed aspirin at diagnosis	3349 (17.0)	1179 (17.3)		5986 (24.5)	2123 (25.2)
Haemoglobin level recorded	15 339 (77.8)	5288 (77.8)		18 490 (75.7)	6269 (74.5)
Platelet count recorded	15 280 (77.5)	5257 (77.3)		18 403 (75.3)	6233 (74.1)
Liver function test result recorded	14 146 (71.8)	4869 (71.6)		17 853 (73.1)	6037 (71.7)
Haemoglobin <110 g/L	5581 (28.3)	1906 (28.0)		4954 (20.3)	1716 (20.4)
Raised platelet count	2321 (11.8)	791 (11.6)		1607 (6.6)	561 (6.7)
Abnormally raised liver function test result	697 (3.5)	229 (3.4)		1188 (4.9)	401 (4.8)
Body mass index (kg/m^2^):					
Recorded	16 368 (83.1)	5620 (82.6)		20 611 (84.3)	7032 (83.6)
<20	1029 (5.2)	342 (5.0)		509 (2.1)	214 (2.5)
20-24.9	5859 (29.7)	2015 (29.6)		6158 (25.2)	2149 (25.5)
25-29.9	5674 (28.8)	1912 (28.1)		9268 (37.9)	3092 (36.7)
30-34.9	2665 (13.5)	924 (13.6)		3636 (14.9)	1236 (14.7)
≥35	1141 (5.8)	427 (6.3)		1040 (4.3)	341 (4.1)

CVD=cardiovascular disease; COPD=chronic obstructive pulmonary disease; VTE=venous thromboembolism.

Baseline characteristics of both men and women were similar in the derivation and validation cohorts. However, there were some differences in baseline characteristics between the sexes. For example, in the QResearch derivation cohort, 69.1% of women had colon cancer compared with 59.7% of men. Use of chemotherapy and radiotherapy was higher in men than in women (33.0% *v* 27.9% and 16.8% *v* 11.8%, respectively). Men were more likely than women to be former smokers (table 2, 40.1% *v* 24.9%), have cardiovascular disease (23.3% *v* 15.5%), and have type 2 diabetes (14.8% *v* 11.2%). Men were also more likely to be prescribed statins (29.7% *v* 21.6%) and aspirin (24.5% *v* 17.0%) at diagnosis. Women were more likely than men to be anaemic at diagnosis (28.3% *v* 20.3%) and have raised platelet count (11.85 *v* 6.6%).

Table 2 in the web appendix compares baseline characteristics of men and women with and without a family history of bowel cancer. Women with a family history tended to be more affluent, have a diagnosis at an earlier stage, and were more likely to have surgery than women without a family history. These differences were less noticeable in men.

### Primary outcomes

In the 44 145 patients in the derivation cohort, there were 26 887 deaths during follow-up, and of these, 13 588 (50.5%) were recorded as due to colorectal cancer. Of the 15 214 patients in the QResearch validation cohort, there were 9404 deaths, and of these, 4770 (50.7%) were recorded as due to colorectal cancer. Of the 437 821 patients in the PHE validation cohort, there were 277 628 deaths. Information on cause of death for patients in the PHE validation cohort was not available.

Table 3 shows the 5 and 10 year age standardised observed and relative net survival values for each cohort. Table 3 also shows the observed and relative net survival since diagnosis conditional on surviving the first year, which shows a noticeable improvement.

**Table 3 tbl3:** Age standardised observed and relative net survival rates for patients with colorectal cancer aged 15-99 years in the QResearch derivation cohort and both validation cohorts. Values are percentages unless stated otherwise

Variables	QResearch derivation cohort			QResearch validation cohort			PHE validation cohort	
	Observed (%)	Relative net survival (95% CI)*		Observed (%)	Relative net survival (95% CI		Observed (%)	Relative net survival (95% CI)
**Since diagnosis**								
5 years:								
Overall	43.5	52.3 (51.7 to 52.9)		42.8	51.5 (50.4 to 52.5)		42.5	51.2 (51.0 to 51.4)
Stage 1	75.0	92.2 (89.8 to 94.1)		76.4	93.4 (88.5 to 96.2)		74.4	91.7 (90.9 to 92.4)
Stage 2	66.1	81.5 (80.1 to 82.8)		64.0	78.6 (76.2 to 80.8)		64.0	79.4 (78.9 to 79.8)
Stage 3	43.7	52.9 (51.5 to 54.2)		42.9	52.3 (49.9 to 54.7)		43.1	52.1 (51.7 to 52.6)
Stage 4	7.9	9.2 (8.3 to 10.1)		8.5	9.8 (8.3 to 11.5)		7.7	8.8 (8.6 to 9.1)
Not recorded	31.2	36.4 (35.4 to 37.4)		30.5	35.7 (34.0 to 37.3)		30.4	35.5 (35.1 to 35.8)
10 years:								
Overall	31.9	49.4 (48.1 to 50.6)		31.8	48.1 (46.3 to 49.9)		30.8	47.3 (47.0 to 47.7)
Stage 1	56.9	91.6 (82.2 to 96.1)		56.2	†		55.6	90.2 (87.3 to 92.5)
Stage 2	48.5	80.2 (75.8 to 83.9)		47.1	76.6 (69.8 to 82.1)		45.9	76.3 (75.1 to 77.6)
Stage 3	30.3	49.0 (45.3 to 52.6)		30.9	47.7 (42.8 to 52.5)		30.0	47.2 (46.2 to 48.3)
Stage 4	4.7	12.1 (7.5 to 17.9)		NS	NS		4.7	6.7 (6.1 to 7.3)
Not recorded	23.5	33.0 (31.5 to 34.6)		23.7	32.3 (30.3 to 34.3)		22.6	31.6 (31.2 to 32.0)
**1 year conditional survival**								
5 years:								
Overall	60.2	71.3 (70.6 to 72.1)		59.9	70.9 (69.5 to 72.2)		59.8	71.1 (70.9 to 71.4)
Stage 1	80.9	96.2 (93.4 to 97.8)		82.6	98.4 (80.4 to 99.9)		80.4	95.7 (94.9 to 96.3)
Stage 2	74.1	88.5 (87.1 to 89.8)		72.3	86.3 (83.7 to 88.6)		72.5	87.2 (86.8 to 87.7)
Stage 3	54.4	64.6 (62.9 to 66.3)		53.9	64.1 (61.2 to 66.9)		54.1	64.2 (63.6 to 64.7)
Stage 4	19.9	23.9 (21.2 to 26.6)		21.9	25.6 (20.8 to 30.7)		19.8	23.0 (22.2 to 23.8)
Not recorded	51.3	59.4 (57.9 to 60.9)		50.9	59.3 (56.8 to 61.7)		51.3	59.8 (59.3 to 60.3)
10 years:								
Overall	42.8	65.9 (63.9 to 67.8)		42.9	64.5 (61.4 to 67.4)		41.8	64.2 (63.6 to 64.8)
Stage 1	60.4	92.3 (83.0 to 96.6)		60.3	†		59.2	90.8 (88.1 to 92.9)
Stage 2	53.4	84.2 (79.4 to 87.9)		52.1	81.5 (73.5 to 87.3)		50.9	81.0 (79.6 to 82.2)
Stage 3	37.0	58.4 (53.5 to 63.1)		37.8	56.5 (50.3 to 62.2)		36.7	56.6 (55.2 to 58.0)
Stage 4	11.4	48.1 (22.0 to 70.3)		NS	NS		11.5	18.6 (15.4 to 22.1)
Not recorded	37.1	52.8 (50.0 to 55.5)		37.5	51.5 (48.2 to 54.7)		36.4	51.8 (51.0 to 52.6)

NS=Not sufficient. Too few events occurred in the QResearch validation cohort to calculate 95% confidence intervals or 10 year survival for stage 4 cancer.

*Relative survival is the ratio of the overall survival for a cohort of patients with cancer to the expected survival in the general population matched by age, sex, and calendar year. Background rates were obtained from the Office for National Statistics. Rates were directly age standardised using standard weights proposed by Corazziari et al^45^: 15-44 (7%), 45-54 (12%), 55-64 (23%), 65-74 (29%), >75% (29%).

†Sample too small to calculate 95% confidence interval.

### Models

#### Colorectal cancer mortality

Table 4 shows the adjusted hazard ratios for deaths from colorectal cancer for variables in the final model for men and women in the derivation cohort. Details of the fractional polynomial terms for age and body mass index and statistically significant interactions are shown in the footnote of table 3 and figure 1. The final models included several variables: age, deprivation, cancer stage, cancer grade, smoking status, colorectal cancer surgery, chemotherapy, family history of bowel cancer, raised platelet count, abnormal liver function test result, cardiovascular disease, diabetes, and prescribed aspirin and statins at diagnosis. In women, increased survival was associated with younger age, colorectal cancer surgery (adjusted hazard ratio 0.47, 95% confidence interval 0.44 to 0.51), family history of bowel cancer (0.58, 0.46 to 0.74), and prescriptions for statins (0.72, 0.66 to 0.78) and aspirin (0.86, 0.77 to 0.96) at diagnosis. The results for men were comparable except for family history where the adjusted hazard ratio for men was 0.82 (0.67 to 0.98). Poorer survival in men and women was associated with a later stage of cancer, a poorly differentiated grade, increasing deprivation, heavy smoking, raised platelet count, abnormally raised liver function test result, diagnosis of cardiovascular disease, and type 2 diabetes. There were statistically significant interactions in men and women between age and cancer stage and between chemotherapy and cancer stage (table 4) and in women between age and aspirin use (fig 1). In women, cancer stage was strongly associated with colorectal cancer mortality. For example, in those who did not have chemotherapy the risk of death from colorectal cancer in those with stage 4 cancer was 35 times higher (adjusted hazard ratio 35.6, 26.5 to 47.9) than women with stage 1 cancer. These hazard ratios were lower in women who had chemotherapy. In men and women with stage 1 and 2 cancer those receiving chemotherapy had an increased risk of colorectal cancer mortality compared with those not receiving chemotherapy, in patients with stage 3 or 4 cancer, chemotherapy was associated with a reduced risk of colorectal cancer mortality. For example, in women with stage 1 cancer the risk of colorectal mortality was 3.2 times higher in those who had chemotherapy than in those who did not have chemotherapy (3.21, 1.95 to 5.31). For women with stage 4 cancer, chemotherapy was associated with a 44% lower risk of death from colorectal cancer (0.56, 0.49 to 0.63).

**Table 4 tbl4:** Adjusted hazard ratios with 95% confidence intervals for death from colorectal cancer in men and women in the derivation cohort for the main model

Predictor variables	Adjusted hazard ratio (95% CI)	
	Women	Men
Townsend score*	1.07 (1.02 to 1.12)	1.05 (1.00 to 1.10)
Smoking status:		
Non-smoker	1.00	1.00
Former smoker	0.93 (0.86 to 1.00)	0.99 (0.93 to 1.05)
Light smoker	1.19 (1.03 to 1.39)	1.33 (1.21 to 1.45)
Moderate smoker	1.16 (0.97 to 1.39)	1.13 (0.97 to 1.32)
Heavy smoker	1.54 (1.28 to 1.87)	1.48 (1.26 to 1.73)
Cancer stage at diagnosis†:		
1	1.00	1.00
2	2.79 (2.13 to 3.65)	2.11 (1.66 to 2.67)
3	10.33 (7.75 to 13.77)	7.48 (6.07 to 9.21)
4	35.63 (26.52 to 47.85)	30.98 (24.87 to 38.59)
Cancer grade at diagnosis:		
1, well differentiated	1.00	1.00
2, moderately differentiated	1.12 (0.94 to 1.33)	1.11 (0.98 to 1.26)
3, poorly differentiated	1.59 (1.33 to 1.90)	1.93 (1.68 to 2.20)
4, undifferentiated	1.65 (0.90 to 3.02)	2.50 (1.26 to 4.95)
Medical history‡:		
Family history of bowel cancer	0.58 (0.46 to 0.74)	0.81 (0.67 to 0.98)
Raised platelet count	1.25 (1.15 to 1.37)	1.28 (1.18 to 1.39)
Abnormal liver function test result	1.35 (1.19 to 1.53)	1.72 (1.56 to 1.89)
Statin use at diagnosis	0.72 (0.66 to 0.78)	0.67 (0.62 to 0.72)
Aspirin use at diagnosis	0.86 (0.77 to 0.96)	0.77 (0.72 to 0.82)
CVD	1.28 (1.16 to 1.41)	1.30 (1.21 to 1.41)
Type 1 diabetes	0.71 (0.35 to 1.44)	1.32 (0.68 to 2.54)
Type 2 diabetes	1.12 (1.02 to 1.23)	1.14 (1.05 to 1.24)
Cancer treatments‡:		
Surgery	0.47 (0.44 to 0.51)	0.54 (0.50 to 0.59)
Chemotherapy (stage 1)	3.21 (1.95 to 5.31)	2.48 (1.64 to 3.74)
Chemotherapy (stage 2)	1.70 (1.39 to 2.07)	1.60 (1.35 to 1.90)
Chemotherapy (stage 3)	0.74 (0.66 to 0.84)	0.77 (0.70 to 0.85)
Chemotherapy (stage 4)	0.56 (0.49 to 0.63)	0.52 (0.48 to 0.57)

CVD=cardiovascular disease.

*Scores range between −7 (most affluent) and 11 (most deprived). Adjusted hazard ratio is per 5 unit increase.

†In people without chemotherapy at the mean age.

‡Adjusted hazard ratio compared with patients without this characteristic. The model for women includes terms for age (two fractional polynomial terms, age^3^ and age^3^ln(age)) and body mass index (two fractional polynomial terms, bmi^−2^ and ln(bmi)). The model for men includes terms for age (two fractional polynomial terms, age^3^ and age^3^ln(age)) and body mass index (two fractional polynomial terms, bmi^0.5^ and bmi). In men and women there were interactions between age and cancer stage and between chemotherapy and cancer stage, and in women there was an interaction between age and aspirin use.

**Figure f1:**
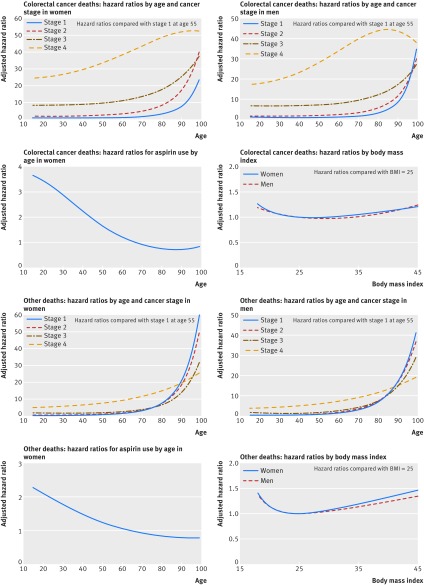
**Fig1** Adjusted hazard ratios for colorectal cancer deaths and other deaths

#### Other cause mortality

Table 5 shows the adjusted hazard ratios for mortality from other causes. The hazard ratios for fractional polynomial terms are shown in figure 1. The model included all the predictors in the colorectal cancer mortality model as well as chronic obstructive pulmonary disease and chronic renal disease. The direction of the hazard ratios was similar to that for the colorectal mortality model, although the magnitude of the hazard ratios was less for cancer grade and stage. In women there was an interaction between age and aspirin use. In men and women there were interactions between age and cancer stage, chemotherapy and cancer stage, cardiovascular disease and cancer stage, and chronic obstructive pulmonary disease and cancer stage. For example, in women with stage 1 cancer, those with cardiovascular disease had a 39% increased risk of other cause mortality compared with those without cardiovascular disease (adjusted hazard ratio 1.39, 95% confidence interval 1.07 to 1.80). Similarly, in women with stage 1 cancer, those with chronic obstructive pulmonary disease had a 111% increased risk of other cause mortality compared with those without chronic obstructive pulmonary disease (2.11, 1.54 to 2.89). The interactions between chemotherapy and cancer stage showed a reduced risk for chemotherapy in people with stage 3 and stage 4 disease. For example, in women with stage 4 cancer those who had chemotherapy had a 49% lower risk of other cause mortality compared with those did not have chemotherapy (0.51, 0.44 to 0.60).

**Table 5 tbl5:** Adjusted hazard ratios with 95% confidence intervals for deaths from other causes in men and women in the derivation cohort for the main model. See footnotes for fractional polynomial terms

Predictor variables	Adjusted hazard ratio (95% CI)	
	Women	Men
Townsend score*	1.14 (1.08 to 1.19)	1.16 (1.11 to 1.20)
Smoking status:		
Non-smoker	1.00	1.00
Former smoker	0.99 (0.92 to 1.06)	1.02 (0.97 to 1.08)
Light smoker	1.57 (1.39 to 1.77)	1.35 (1.23 to 1.49)
Moderate smoker	1.52 (1.31 to 1.76)	1.73 (1.46 to 2.05)
Heavy smoker	1.52 (1.18 to 1.97)	1.63 (1.38 to 1.93)
Cancer stage at diagnosis†:		
1	1.00	1.00
2	1.24 (1.06 to 1.46)	1.21 (1.08 to 1.37)
3	2.14 (1.78 to 2.57)	2.08 (1.82 to 2.39)
4	8.53 (7.11 to 10.24)	6.76 (5.72 to 7.98)
Cancer grade at diagnosis:		
1, well differentiated	1.00	1.00
2, moderately differentiated	0.96 (0.84 to 1.10)	1.03 (0.93 to 1.15)
3, poorly differentiated	1.27 (1.07 to 1.51)	1.23 (1.08 to 1.39)
4, undifferentiated	0.84 (0.39 to 1.81)	2.07 (0.89 to 4.83)
Medical history‡:		
Family history of bowel cancer	0.66 (0.54 to 0.80)	0.84 (0.69 to 1.03)
Raised platelet count	1.04 (0.95 to 1.13)	1.15 (1.05 to 1.27)
Abnormally raised liver function test result	1.36 (1.16 to 1.59)	1.37 (1.22 to 1.54)
Statins use at diagnosis	0.84 (0.78 to 0.91)	0.82 (0.78 to 0.87)
Aspirin use at diagnosis	0.90 (0.81 to 1.00)	0.82 (0.78 to 0.88)
CVD (stage 1)	1.39 (1.07 to 1.80)	1.67 (1.42 to 1.96)
CVD (stage 2)	1.01 (0.74 to 1.38)	0.86 (0.70 to 1.04)
CVD (stage 3)	1.05 (0.76 to 1.44)	0.77 (0.64 to 0.94)
CVD (stage 4)	0.82 (0.59 to 1.14)	0.74 (0.60 to 0.90)
Type 1 diabetes	1.60 (0.92 to 2.79)	1.65 (0.90 to 3.03)
Type 2 diabetes	1.23 (1.12 to 1.34)	1.24 (1.16 to 1.33)
Renal disease	1.65 (1.39 to 1.96)	1.55 (1.29 to 1.86)
COPD (stage 1)	2.11 (1.54 to 2.89)	1.63 (1.33 to 2.00)
COPD (stage 2)	0.71 (0.46 to 1.11)	1.10 (0.86 to 1.41)
COPD (stage 3)	0.84 (0.57 to 1.23)	0.98 (0.77 to 1.24)
COPD (stage 4)	0.54 (0.36 to 0.80)	0.75 (0.56 to 0.99)
Cancer treatments‡:		
Surgery	0.63 (0.58 to 0.69)	0.62 (0.58 to 0.67)
Chemotherapy (stage 1)	1.42 (0.96 to 2.11)	0.93 (0.69 to 1.27)
Chemotherapy (stage 2)	0.94 (0.78 to 1.12)	0.96 (0.85 to 1.10)
Chemotherapy (stage 3)	0.69 (0.60 to 0.79)	0.69 (0.62 to 0.76)
Chemotherapy (stage4)	0.51 (0.44 to 0.60)	0.53 (0.45 to 0.61)

CVD=cardiovascular disease; COPD=chronic obstructive pulmonary disease.

*Scores range between −7 (most affluent) and 11 (most deprived). Adjusted hazard ratio is per 5 unit increase.

†In people without chemotherapy and without CVD and without COPD at the mean age.

‡Adjusted hazard ratio compared with patients without this characteristic. Model for women includes terms for age (two fractional polynomial terms, age^2^ and age^2^ln(age)) and body mass index (two fractional polynomial terms, bmi^−2^ and bmi^−2^ln(bmi)). The model for men includes terms for age (two fractional polynomial terms, age and ageln(age)) and body mass index (two fractional polynomial terms, bmi^−2^ and bmi^−2^ln(bmi)). In men and women there were interactions between age and cancer stage, chemotherapy and cancer stage, CVD and cancer stage, and COPD and cancer stage. In women there was an interaction between age and aspirin use.

#### Landmark analysis

We undertook a landmark analysis, restricted to the 13 926 women and 17 935 men who survived the first year after diagnosis. The results for colorectal mortality are shown in table 3 in the web appendix. The adjusted hazard ratio for colorectal surgery in women was 0.65 (95% confidence interval 0.59 to 0.71) and for chemotherapy (stage 1 cancer) was 1.97 (1.40 to 2.75). The corresponding figures in men were 0.62 (0.56 to 0.67) and 1.71 (1.34 to 2.16). In men and women with stage 3 and 4 cancer, chemotherapy was not associated with a reduced risk of death from colorectal cancer.

### Validation—discrimination and calibration

Table 6 shows the performance of each equation in both validation cohorts for five year survival estimates. Table 4 in the web appendix shows the corresponding results at 1 and 10 years. For example, in women the five year equation for colorectal cancer mortality explained 45.3% of the variation in the QResearch validation cohort; the D statistic was 1.86 and Harrell’s C statistic was 0.80. The corresponding results for all cause mortality were 42.6%; 1.77 and 0.79. Performance for one year risk estimates was marginally better and those for 10 year estimates were marginally worse (see table 4 in the web appendix). Overall, performance in the QResearch validation cohort was marginally better than that in the PHE cohort for all comparisons in both men and women and at all time points.

**Table 6 tbl6:** Performance of the equations in men and women for all cause mortality and colorectal cancer mortality in QResearch validation cohort and Public Health England (PHE) cancer registry validation cohort at five year survival

Outcome and statistic	Women			Men	
	QResearch cohort	PHE cohort		QResearch cohort	PHE cohort
All cause mortality:					
D	1.765 (1.681 to 1.849)	1.656 (1.637 to 1.676)		1.711 (1.651 to 1.771)	1.603 (1.586 to 1.620)
R^2^ (%)	42.643 (40.322 to 44.965)	39.579 (39.030 to 40.129)		41.144 (39.447 to 42.841)	38.024 (37.523 to 38.525)
Harrell’s C	0.787 (0.779 to 0.795)	0.775 (0.773 to 0.776)		0.780 (0.773 to 0.788)	0.763 (0.762 to 0.764)
Colorectal mortality:					
D	1.862 (1.758 to 1.966)	NA		1.869 (1.765 to 1.972)	NA
R^2^ (%)	45.273 (42.507 to 48.038)	NA		45.455 (42.729 to 48.182)	NA
Harrell’s C	0.797 (0.784 to 0.810)	NA		0.800 (0.791 to 0.809)	NA

NA=not available.

Figure 2 shows the mean predicted risks and observed risks by 10th of predicted risk at 1, 5, and 10 years for men and women for all cause mortality in both validation cohorts as well as the corresponding graphs for colorectal cancer mortality in the QResearch validation cohort. The correspondence was close between the mean predicted risks and the observed risks, indicating that the equations were well calibrated for each of the time points and across both validation cohorts and for both outcomes.

**Figure f2:**
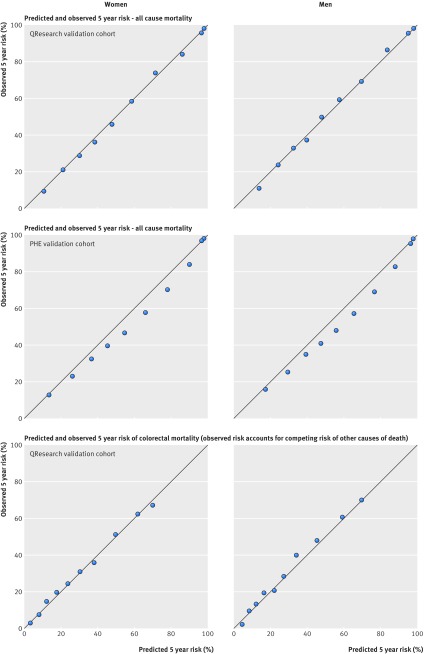
**Fig 2** Calibration plots for all cause mortality and colorectal cancer mortality. PHE=Public Health England

### Decision curve analysis

Figure 3 displays the net benefit curves for both colorectal cancer mortality and other cause mortality equations at five years in men and women. The prediction equations for colorectal mortality and for other cause mortality had higher net benefit than strategies based on considering either no patients or all patients for intervention across a range of risk thresholds.

**Figure f3:**
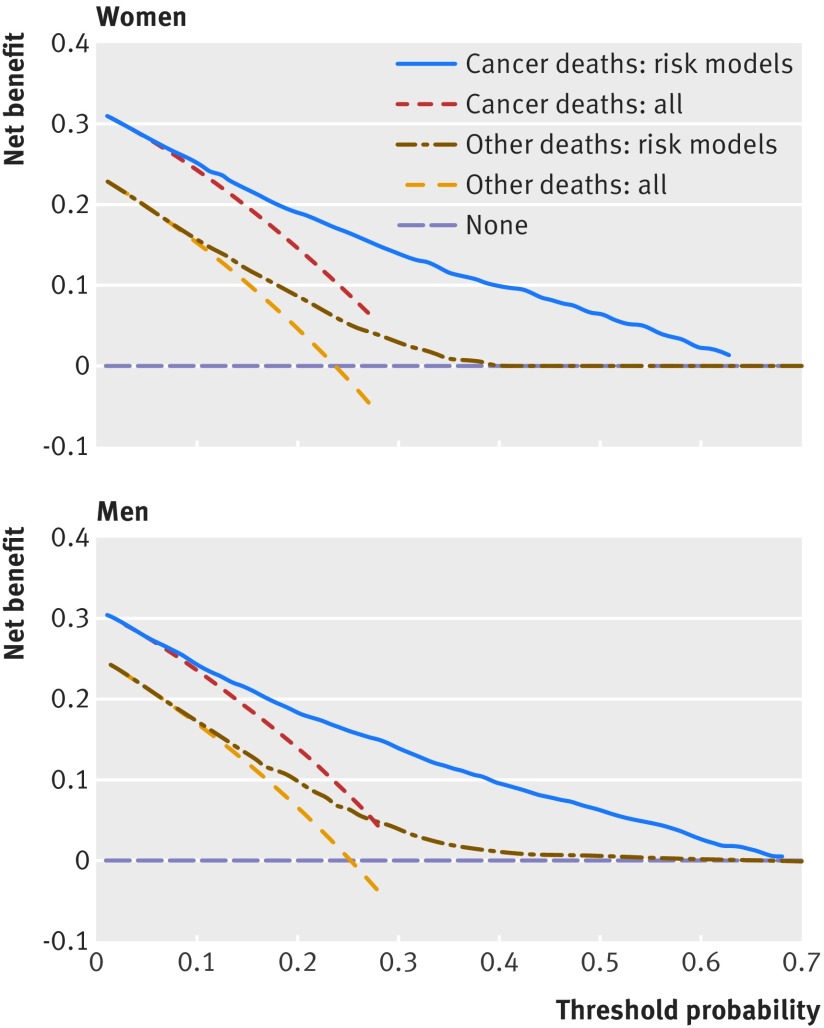
**Fig 3** Decision curves for five year risk in women and men

### Web calculator

Figures 4 and 5[Fig f4 f5] show a clinical example of the implementation of the equations as a web calculator (http://qcancer.org/colorectal-survival/index.php), illustrating how the number of years since diagnosis affects survival estimates. The example in figure 4 is for a 38 year old woman with grade 1, stage 4 colorectal cancer who has had a hemicolectomy and chemotherapy. Her initial five year survival estimate at diagnosis is 45.0% (white bar), risk of death from colorectal cancer is 35.6% (blue bar), and risk of death from other causes is 19.4% (dark blue bar). Having survived for 12 months since diagnosis, her five year survival estimate is 57.1%, her five year risk of death from colorectal cancer is 25.8%, and her five year risk of death from other causes is 17.0% (fig 5).

**Figure f4:**
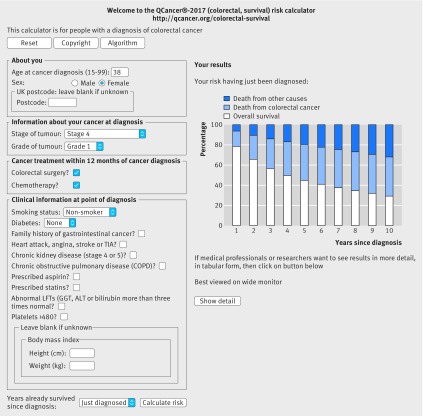
**Fig 4** Web calculator showing survival estimates for clinical example 1 at diagnosis

**Figure f5:**
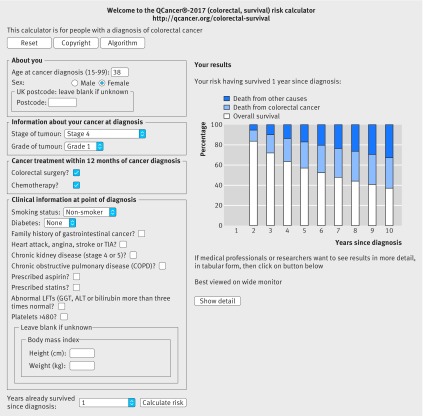
**Fig 5** Web calculator showing survival estimates for clinical example 1, 12 months after diagnosis

Figures 6 and 7[Fig f6 f7] show a second example, illustrating how the general practice derived variables affect survival estimates. The example in figure 6 is for a 65 year old woman with grade 3, stage 2, colorectal cancer who is a non-smoker. Her five year survival estimate at diagnosis is 63.1% (white bar), five year risk of death from colorectal cancer is 21.6% (blue bar), and risk of death from other causes is 15.3% (dark blue bar). If she is a heavy smoker, has stage 4 or 5 kidney disease, an abnormal liver function test result, and raised platelet count, her five year survival estimate is 24.9%, her five year risk of death from colorectal cancer is 39.5%, and her five year risk of death from other causes is 35.6% (fig 7).

**Figure f6:**
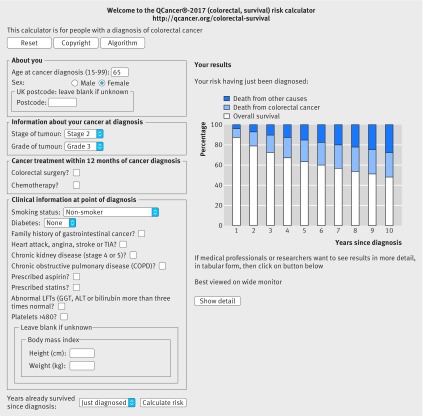
**Fig 6** Web calculator showing survival estimates for clinical example 2, based on cancer registration data alone

**Figure f7:**
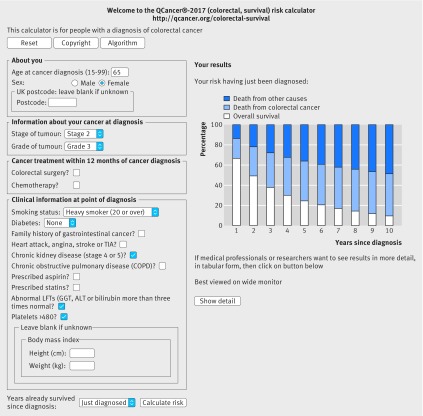
**Fig 7** Web calculator showing survival estimates for clinical example 2, including both cancer registration data and general practice derived data

Figure 8 shows a third example, illustrating how the competing risk of death from other causes (dark blue bar) becomes the predominant factor over time, especially in elderly people. The example in figure 8 is for an 80 year old woman with stage 1, grade 1 colorectal cancer who is a light smoker and has angina. Her estimated overall survival at one year (white bar) is 75.1%, at five years is 32.2%, and at 10 years is 10.8%. Her risk of death from colorectal cancer (blue bar) at corresponding time points is 6.2%, 13.5%, and 14.3%. Her mortality from other causes (dark blue bar) at these time points is 18.4%, 53.9%, and 74.5%. The web calculator can then be used to show the risks if she has colorectal surgery and/or chemotherapy to help inform her decisions about treatments.

**Figure f8:**
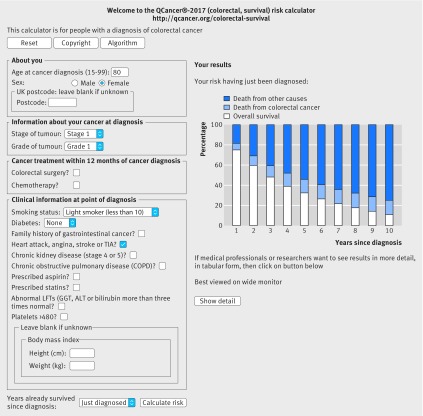
**Fig 8** Web calculator showing survival estimates for clinical example 3, illustrating how the competing risk of death from other causes can become the predominant factor over time

## Discussion

We have developed and externally validated equations to predict the absolute risks of both all cause mortality and colorectal cancer specific mortality for men and women with colorectal cancer over 1, 5, and 10 years. The equations are well calibrated and have good discrimination, with Harrell’s C statistics at least 0.76 in both validation cohorts for both outcomes (all cause mortality and colorectal cancer mortality). These models are designed to provide better information on survival for individual patients, taking account of their profile, including both conventional factors (age and cancer stage) and deprivation index, smoking status, common comorbidities, prescribed drugs, and cancer treatments such as surgery and chemotherapy. These models, based on information likely to be available at the point of diagnosis, have been designed to help improve the management decisions by patients and clinicians in primary and secondary care.

We have provided a web based calculator (http://qcancer.org/colorectal-survival/index.php) to illustrate how the risks vary with the different predictors and over different time periods. The equations to predict absolute risk of death from colorectal cancer account for competing risk of death from other causes. This is useful for identifying patients with a high risk of death from colorectal cancer but with an otherwise low risk of death from other causes for whom more aggressive treatment may be appropriate. Equally they can identify patients with a low risk of death from colorectal cancer and a high risk of death from other causes for whom palliative care may be more appropriate. However, management plans would need to be based on joint decisions between clinicians and patients, reflecting individual thoughts around risks and benefits of treatment or concerns about side effects, living circumstance, or support available, rather than prespecified cut-offs.

We have also provided conditional survival estimates, which incorporate how risks change over time. These are particularly important among patients where the initial prognosis is poor owing to late stage disease. For example, a 38 year old woman who has just received a diagnosis of stage 4 well differentiated colorectal cancer may want to know her estimated survival with and without a hemicolectomy and chemotherapy to help her assess the potential value of surgery. Using the web calculator, her five year survival estimate would be 5.9% without either colorectal surgery or chemotherapy. With colorectal surgery alone it would be 22.6% and with both treatments it would be 44.8%. This compares with the published five year survival estimate of 66% based solely on her age (which is an overestimate) or the 8% based solely on her cancer stage (which is an underestimate if she receives treatment).^2^ Assuming she has both colorectal surgery and chemotherapy and survives for a year after diagnosis, her five year conditional survival after diagnosis would have increased to 57.1%. Instead of the currently available static prediction based on one or two variables, she would be able to obtain dynamic predictions, which can be updated for each successive year after her original diagnosis.^31^ More accurate dynamic survival estimates are particularly valuable for patients who need to make important life decisions and whose quality of life may be affected by uncertainty about the future. For treating doctors, dynamic conditional survival could help with planning the optimum duration and intensity of follow-up.^10^ Conditional survival estimates could also be used to stratify patients on entry to clinical trials. The time at which the conditional survival plateaus has been advocated as a potentially important endpoint in clinical trial design (eg, the survival for head and neck cancer plateaus at three years, leading to suggestions that three year clinical trial data may provide a valid endpoint).^46^

### Comparisons with the literature

Our study builds on several original studies that have developed colorectal cancer prognostic models to predict overall survival based on patients recruited to clinical trials in USA^5^ or US cancer registries.^4^ ^6^ In contrast with other studies, our model predicts both overall survival and colorectal cancer specific survival. It also predicts survival over a longer period (10 years rather than five years). Our model provides conditional survival predictions that can be updated for each additional year of survival since the original cancer diagnosis. It does not, however, predict recurrence-free survival unlike other models,^4^ ^5^ as the date of recurrence is not well recorded on our dataset.

Our model takes account of more variables than other models.^4^ ^5^ ^6^ The model by Renfo et al^5^ includes age, sex, race, body mass index, performance status, cancer grade, cancer stage, ratio of positive lymph nodes to nodes examined, number and location of primary cancers, and chemotherapy. It can only be applied to those with stage 3 cancer. The model by Weiser et al includes cancer stage, cancer grade, number of lymph nodes examined, number of positive lymph nodes, age, and sex.^6^ It can be applied to patients who have curative intent surgery. A third model, Adjuvant online^4^ includes cancer stage, cancer grade, 10 year age band, sex, and comorbidity (perfect health, minor problems, average for age, or major problems). It can be applied to patients with stage 2 or 3 colon cancer. It has been validated although the results focused on calibration rather than on discrimination.^3^ The Adjuvant online model itself has not been published and the website is currently unavailable.

Our models have been developed using a large, representative population based cohort and can be applied to all patients with a first diagnosis of colorectal cancer including all stages of disease and those who have or have not had chemotherapy. Our model has better discrimination than previous models, with Harrell’s C statistic of 0.76 to 0.78 compared with 0.66^5^ to 0.68.^6^ As with the Weiser’s model, our model was well calibrated, whereas the Adjuvant online model tended to overestimate survival for patients with stage 2 disease.^3^

We included established predictors in our equations and report hazard ratios similar in both magnitude and direction to those reported elsewhere, increasing the clinical validity. For example, the 17-18% reduced all cause mortality for patients prescribed aspirin at diagnosis of colorectal cancer is consistent with studies from Norway and the Netherlands.^20^ ^21^ Similarly, the 23-29% reduced mortality among those prescribed statins at diagnosis is similar to that reported in a Danish study.^19^ The observed and relative net survival rates overall and by cancer stage are comparable to those reported elsewhere.^14^ ^38^ For example, the relative net five year survival in the Public Health England (PHE) validation cohort was 51.2% and the conditional value was 71.1% after one year. Coleman et al reported corresponding values for England (2005-07) of 53.7% and 71.8%.^14^

We found some evidence of a beneficial effect of chemotherapy for patients with stages 3 and 4 disease, with reductions in colorectal cancer mortality of between 32% and 49%, which is broadly consistent with observational studies^47^ and meta-analyses of clinical trials.^48^ However, we found no clear evidence of a beneficial effect of chemotherapy for patients with stages 1 and 2 disease, although the numbers of patients receiving treatment were relatively small and our study was an observational one rather than a randomised controlled clinical trial so subject to residual confounding. However, our model could also be used to risk stratify patients recruited to clinical trials, as has been proposed by Weiser et al for similar US based algorithms.^6^ For example, if higher risk groups can be identified, then the sample size needed to show a defined benefit could be reduced. This could be useful for trials of chemotherapy in patients with stage 1 and 2 disease where the benefit of chemotherapy is less certain.

### Methodological considerations

The statistical methods we have used to derive and validate these models are similar to those for other risk prediction tools derived from the QResearch database, the strengths and limitations of which have been discussed in detail.^11^ ^49^ In summary, key strengths include cohort size, duration of follow-up, representativeness, and lack of selection, recall, and respondent bias. UK general practices have good levels of accuracy and completeness in recording clinical diagnoses and prescribed drugs.^50^ The QResearch database has comprehensive linked cancer and mortality records for virtually all patients and is therefore likely to have picked up most cases of colorectal cancer and related deaths, thereby minimising ascertainment bias.

We decided to present separate models for men and women in our protocol since there were likely to be important differences in baseline characteristics between the sexes. Our analysis showed differences for tumour location (women were more likely to have colon cancer than men), uptake of chemotherapy and radiotherapy (which was lower in women than men), and baseline characteristics (men were more likely to smoke, have cardiovascular disease or diabetes, and be prescribed aspirin and statins at baseline). We also found that in women a family history of bowel cancer was associated with improved survival, but this was less so in men. Women with a family history of bowel cancer tended to be more affluent and also to receive a diagnosis at an earlier stage than women without a family history of bowel cancer (table 2 in the web appendix). A possible explanation for these findings may be that women with a family history are more aware of red flag symptoms and present sooner to their general practitioner and are referred more quickly. However, we did include both deprivation and cancer stage in our multivariate model.

We undertook two validations, one using a separate set of practices and patients contributing to QResearch and the other using patients not registered with QResearch practices but included on the PHE cancer registry. The results of both validations were similar, suggesting that the results are likely to be generalisable to the population of England. The QResearch validation cohort included all the variables for men and women used to derive the scores (age, body mass index, Townsend score, cancer stage, cancer grade, colorectal surgery, family history, raised platelet count, abnormal liver function test result, statin use, aspirin use, cardiovascular disease, diabetes, renal disease, and chronic obstructive pulmonary disease). The fully external NCRAS cohort was limited to the following variables: age, cancer grade, cancer age, Townsend score, colorectal surgery, and chemotherapy. This may explain why the performance was marginally worse in the NCRAS validation cohort (table 6).

Other limitations of our study include the lack of formal adjudication of diagnoses, although the accuracy of cancer diagnoses is likely to be good given the development and utility of the underlying NCRAS dataset and its use for national statistics, as well as for multiple international research studies.^14^ ^38^ Our primary outcome was death from all causes and we are confident that the date of death will be reliable since we have used national death certification information. Our secondary outcome was death from colorectal cancer and this may be susceptible to misclassification bias since not all patients will have a post-mortem examination. Hence it is possible that some patients died of colorectal cancer but had another cause of death recorded and vice versa, which could lead to some bias in estimates of cancer specific survival.^51^

There is also potential for bias for predictor variables owing to missing data for cancer stage, cancer grade, smoking status, and body mass index, although this was addressed using multiple imputation. Dates of colorectal surgery and chemotherapy were unavailable on our subset of the NCRAS dataset, other than whether they occurred within 12 months of the date of diagnosis. This could lead to a survival bias,^32^ ^33^ making colorectal surgery appear to be unduly effective, although our additional landmark analysis suggests this is likely to be marginal. Other potential variables that we have not incorporated include the timeliness of treatments and the availability of surveillance lower endoscopy examinations. Geographical accessibility of medical service providers may also impact on regular treatment and surveillance of patients with colorectal cancer.

While we have derived and validated the equations using English datasets, the equations could apply internationally by using alternative deprivation scores relevant to the setting. Alternatively the postcode (and hence deprivation scores) can be omitted in the web calculator in which case a value of zero will be assumed in the calculations. The result of this will be to overestimate survival among deprived patients and underestimate it for more affluent patients. Local validation should be done to ensure good calibration and discrimination in the applicable population.

### Conclusions

We have developed and validated new risk prediction equations to quantify overall and cancer specific survival of patients with colorectal cancer, taking account of an individual’s clinical and demographic characteristics. A more individualised approach to prognosis will help improve the accuracy of information for patients and hence decision making. It could also assist with informing follow-up schedules.

**What is already known on this topic**

Realistic estimates of overall survival are important for patients with a diagnosis of colorectal cancer who need to make decisions about the risks and benefits of surgery, chemotherapy, radiotherapy, or palliative care
There is a lack of robust information on survival that takes account of patient characteristics and the likely effect of different treatments
Prognostic models that include more variables tend to produce more accurate predictions than those simply based on stage of cancer at diagnosis

**What this study adds**

New prognostic models for colorectal cancer that predict both overall survival and colorectal mortality were developed and validated
The models include the facility to update the survival estimates conditional on the number of years of survival since diagnosis
Compared with other models, they predict survival over a longer period and have better discrimination

## Web Extra

Extra material supplied by the author

## Notes

A web calculator to calculate estimates of absolute survival can be accessed at http://qcancer.org/colorectal-survival/index.php/. Open source software is also available for download. We thank the EMIS practices who contribute to QResearch; EMIS for expertise in establishing, developing, and supporting the database; Public Health England (PHE) for supplying the cancer registration data; and the Office of National Statistics (ONS) for providing the mortality data. PHE and ONS bear no responsibility for the analysis or interpretation of the data.

Ethical approval: This study was reviewed in accordance with the QResearch agreement with National Research Ethics Service East Midlands, Derby (reference 03/4/021).

Data sharing: The equations presented in this paper will be released as open source software under the GNU lesser General Public License v3. The open source software allows use without charge under the terms of the GNU lesser public license version 3. Closed source software can be licensed at a fee.

Transparency: The manuscript’s guarantor (JHC) affirms that the manuscript is an honest, accurate, and transparent account of the study being reported; that no important aspects of the study have been omitted; and that any discrepancies from the study as planned (and, if relevant, registered) have been explained.
