# Supplementary material for: Development and validation of risk prediction equations to estimate survival in patients with colorectal cancer: cohort study
Source: BMJ. 2017 Jun 15;357:j2497. doi: 10.1136/bmj.j2497 (PMC5471851; doi:10.1136/bmj.j2497)
Supplement: Supplementary file 1 — Appendix: Supplementary materials [file hipj035185.ww.pdf]

Supplementary table 1 comparison of predictor variables in patients with complete data compared with those with one or more missing values.

	QResearch derivation cohort		*QResearch validation cohort*	
	**complete data**	**1+ missing value**	**complete data**	**1+ missing value**
total patients	17642	26503	5716	9498
men	9869 (55.9)	14568 (55.0)	3166 (55.4)	5248 (55.3)
**age in years at diagnosis**				
mean(SD)	71.3 (10.9)	71.9 (12.9)	71.3 (11.1)	72.0 (12.8)
15-19 years	1 (0.0)	22 (0.1)	1 (0.0)	6 (0.1)
20-29 years	27 (0.2)	105 (0.4)	8 (0.1)	41 (0.4)
30-39 years	132 (0.7)	324 (1.2)	53 (0.9)	108 (1.1)
40-49 years	527 (3.0)	1009 (3.8)	182 (3.2)	335 (3.5)
50-59 years	1783 (10.1)	2856 (10.8)	576 (10.1)	1005 (10.6)
60-69 years	4454 (25.2)	6019 (22.7)	1388 (24.3)	2217 (23.3)
70-79 years	6439 (36.5)	7986 (30.1)	2095 (36.7)	2843 (29.9)
80-89 years	3959 (22.4)	6669 (25.2)	1318 (23.1)	2395 (25.2)
90+ years	320 (1.8)	1513 (5.7)	95 (1.7)	548 (5.8)
**Townsend deprivation score (in fifths)**				
most affluent	4216 (23.9)	5938 (22.4)	1397 (24.4)	2273 (23.9)
2	4304 (24.4)	6083 (23.0)	1276 (22.3)	1972 (20.8)
3	3590 (20.3)	5215 (19.7)	1156 (20.2)	1899 (20.0)
4	3056 (17.3)	4739 (17.9)	1021 (17.9)	1738 (18.3)
most deprived	2476 (14.0)	4528 (17.1)	866 (15.2)	1616 (17.0)
**Stage**				
stage recorded	17642 (100.0)	14595 (55.1)	5716 (100.0)	4926 (51.9)
stage 1	2673 (15.2)	2120 (8.0)	882 (15.4)	753 (7.9)
stage 2	6246 (35.4)	4463 (16.8)	2009 (35.1)	1525 (16.1)
stage 3	6195 (35.1)	4725 (17.8)	2051 (35.9)	1646 (17.3)
stage 4	2528 (14.3)	3287 (12.4)	774 (13.5)	1002 (10.5)
**Grade**				
grade recorded	17642 (100.0)	16728 (63.1)	5716 (100.0)	5858 (61.7)
well differentiated	1060 (6.0)	1356 (5.1)	332 (5.8)	513 (5.4)
moderately different	13541 (76.8)	12624 (47.6)	4466 (78.1)	4443 (46.8)
Poorly differentiate	3012 (17.1)	2714 (10.2)	911 (15.9)	882 (9.3)
undifferentiated	29 (0.2)	34 (0.1)	7 (0.1)	20 (0.2)
smoking status recorded	17642 (100.0)	23807 (89.8)	5716 (100.0)	8540 (89.9)
non-smoker	9065 (51.4)	12466 (47.0)	2962 (51.8)	4412 (46.5)
ex-smoker	6729 (38.1)	7975 (30.1)	2162 (37.8)	2876 (30.3)

light smoker	1131 (6.4)	2058 (7.8)	364 (6.4)	762 (8.0)
moderate smoker	438 (2.5)	766 (2.9)	131 (2.3)	274 (2.9)
heavy smoker	279 (1.6)	542 (2.0)	97 (1.7)	216 (2.3)
Missing values±				
1 value missing only	0	10408 (39.3)	0	3604 (37.9)
2 values missing only	0	9032 (34.1)	0	3233 (34.0)
3 values missing only	0	3807 (14.4)	0	1426 (15.0)
4 values missing only	0	2312 (8.7)	0	870 (9.2)
5 values missing only	0	652 (2.5)	0	254 (2.7)
6 values missing	0	292 (1.1)	0	111 (1.2)
not recorded	0	7166 (27.0)	0	2562 (27.0)
< 20	610 (3.5)	928 (3.5)	200 (3.5)	356 (3.7)
20 to 24.9	5358 (30.4)	6659 (25.1)	1769 (30.9)	2395 (25.2)
25-29.9	7275 (41.2)	7667 (28.9)	2342 (41.0)	2662 (28.0)
30-34.9	3255 (18.5)	3046 (11.5)	1026 (17.9)	1134 (11.9)
35+	1144 (6.5)	1037 (3.9)	379 (6.6)	389 (4.1)
**cancer treatments**				
surgery	15938 (90.3)	17330 (65.4)	5192 (90.8)	6055 (63.8)
chemotherapy	6368 (36.1)	7199 (27.2)	1977 (34.6)	2537 (26.7)
**Medical history**				
family history of bowel cancer	467 (2.6)	528 (2.0)	154 (2.7)	191 (2.0)
cardiovascular disease	3855 (21.9)	4900 (18.5)	1281 (22.4)	1770 (18.6)
type 1 diabetes	47 (0.3)	50 (0.2)	17 (0.3)	23 (0.2)
type 2 diabetes	2990 (16.9)	2834 (10.7)	966 (16.9)	1036 (10.9)
chronic renal disease	242 (1.4)	367 (1.4)	65 (1.1)	137 (1.4)
flag	1168 (6.6)	1797 (6.8)	416 (7.3)	655 (6.9)
prescribed statins at diagnosis	6453 (36.6)	5051 (19.1)	2043 (35.7)	1809 (19.0)
prescribed aspirin at diagnosis	4482 (25.4)	4853 (18.3)	1495 (26.2)	1807 (19.0)
BMI recorded	17642 (100.0)	19337 (73.0)	5716 (100.0)	6936 (73.0)
platelets recorded	17642 (100.0)	16041 (60.5)	5716 (100.0)	5774 (60.8)
LFTs recorded	17642 (100.0)	14357 (54.2)	5716 (100.0)	5190 (54.6)
raised platelets	1885 (10.7)	2043 (7.7)	565 (9.9)	787 (8.3)
raised LFTs	814 (4.6)	1071 (4.0)	262 (4.6)	368 (3.9)

± missing values for 6 variables in the final prediction models – stage, grade, smoking status body mass index, LFTs, platelets

Supplementary Table 2: characteristics of men and women in the derivation cohort with and without a family history of bowel cancer.

	women	*women*	*men*	*men*
	**no FH**	**FH**	**no FH**	**FH**
Total cases	19194	514	23956	481
Mean age at diagnosis (SD)	72.6 (12.8)	68.2 (12.7)	71.0 (11.5)	66.3 (12.1)
colon cancer	13282 (69.2)	359 (69.8)	14275 (59.6)	304 (63.2)
rectal cancer	4618 (24.1)	117 (22.8)	7802 (32.6)	137 (28.5)
recto-sigmoid	1294 (6.7)	38 (7.4)	1879 (7.8)	40 (8.3)
most affluent	4342 (22.6)	163 (31.7)	5517 (23.0)	132 (27.4)
2	4492 (23.4)	120 (23.3)	5649 (23.6)	126 (26.2)
3	3868 (20.2)	100 (19.5)	4756 (19.9)	81 (16.8)
4	3495 (18.2)	64 (12.5)	4168 (17.4)	68 (14.1)
most deprived	2997 (15.6)	67 (13.0)	3866 (16.1)	74 (15.4)
stage recorded	13787 (71.8)	406 (79.0)	17670 (73.8)	374 (77.8)
stage 1	1947 (10.1)	83 (16.1)	2697 (11.3)	66 (13.7)
stage 2	4791 (25.0)	135 (26.3)	5669 (23.7)	114 (23.7)
stage 3	4582 (23.9)	133 (25.9)	6075 (25.4)	130 (27.0)
stage 4	2467 (12.9)	55 (10.7)	3229 (13.5)	64 (13.3)
grade recorded	14645 (76.3)	432 (84.0)	18907 (78.9)	386 (80.2)
well differentiated	1075 (5.6)	35 (6.8)	1277 (5.3)	29 (6.0)
moderately different	10841 (56.5)	321 (62.5)	14712 (61.4)	291 (60.5)
Poorly differentiate	2701 (14.1)	76 (14.8)	2883 (12.0)	66 (13.7)
undifferentiated	28 (0.1)	0 (0.0)	35 (0.1)	0 (0.0)
surgery	14300 (74.5)	429 (83.5)	18164 (75.8)	375 (78.0)
chemotherapy	5331 (27.8)	173 (33.7)	7865 (32.8)	198 (41.2)
radiotherapy	2271 (11.8)	54 (10.5)	4037 (16.9)	79 (16.4)
smoking status recorded	17905 (93.3)	500 (97.3)	22571 (94.2)	473 (98.3)
Non-smoker	11122 (57.9)	294 (57.2)	9921 (41.4)	194 (40.3)
Ex-smoker	4762 (24.8)	136 (26.5)	9591 (40.0)	215 (44.7)
light smoker	1154 (6.0)	41 (8.0)	1955 (8.2)	39 (8.1)
moderate smoker	572 (3.0)	12 (2.3)	608 (2.5)	12 (2.5)
heavy smoker	295 (1.5)	17 (3.3)	496 (2.1)	13 (2.7)
Medical history				
other cancer	1518 (7.9)	52 (10.1)	1876 (7.8)	36 (7.5)
cardiovascular disease	2990 (15.6)	64 (12.5)	5612 (23.4)	89 (18.5)
type 1 diabetes	28 (0.1)	1 (0.2)	67 (0.3)	1 (0.2)
type 2 diabetes	2166 (11.3)	41 (8.0)	3552 (14.8)	65 (13.5)

chronic renal disease	263 (1.4)	3 (0.6)	339 (1.4)	4 (0.8)
chronic liver disease	139 (0.7)	4 (0.8)	232 (1.0)	10 (2.1)
inflammatory bowel disease	294 (1.5)	7 (1.4)	359 (1.5)	7 (1.5)
COPD	1007 (5.2)	28 (5.4)	1902 (7.9)	28 (5.8)
venous thromboembolism	886 (4.6)	15 (2.9)	925 (3.9)	14 (2.9)
statins at diagnosis	4132 (21.5)	126 (24.5)	7097 (29.6)	149 (31.0)
aspirin at diagnosis	3253 (16.9)	96 (18.7)	5882 (24.6)	104 (21.6)
Haemoglobin recorded	14915 (77.7)	424 (82.5)	18114 (75.6)	376 (78.2)
platelets recorded	14855 (77.4)	425 (82.7)	18029 (75.3)	374 (77.8)
HB < 11 g/DL	5464 (28.5)	117 (22.8)	4871 (20.3)	83 (17.3)
raised platelets	2266 (11.8)	55 (10.7)	1572 (6.6)	35 (7.3)
raised LFTs	682 (3.6)	15 (2.9)	1169 (4.9)	19 (4.0)
BMI recorded	15899 (82.8)	469 (91.2)	20172 (84.2)	439 (91.3)
< 20 kg/m^2	1004 (5.2)	25 (4.9)	500 (2.1)	9 (1.9)
20 to 24.9 kg/m^2	5692 (29.7)	167 (32.5)	6046 (25.2)	112 (23.3)
25-29.9 kg/m^2	5504 (28.7)	170 (33.1)	9061 (37.8)	207 (43.0)
30-34.9 kg/m^2	2600 (13.5)	65 (12.6)	3548 (14.8)	88 (18.3)
35+ kg/m^2	1099 (5.7)	42 (8.2)	1017 (4.2)	23 (4.8)

Supplementary Table 3 Adjusted hazard ratios with 95% confidence intervals for colorectal cancer death in men and women in the derivation cohort for the main model restricted to those surviving the first year (landmark analysis). For fractional polynomial terms see footnotes and figure 1.

	Adjusted hazard ratio (95% CI)	***Adjusted hazard ratio (95% CI)***
	**women**	**men**
Townsend Score$	1.10 (1.05 to 1.15)	1.10 (1.06 to 1.15)
**Smoking status**		
non-smoker	1.00	1.00
ex-smoker	0.98 (0.92 to 1.05)	1.04 (0.98 to 1.10)
light smoker	1.32 (1.17 to 1.50)	1.41 (1.27 to 1.57)
moderate smoker	1.37 (1.18 to 1.60)	1.54 (1.33 to 1.78)
heavy smoker	1.49 (1.15 to 1.91)	1.72 (1.45 to 2.04)
**Stage at diagnosis±**		
stage 1	1.00	1.00
stage 2	1.32 (1.16 to 1.50)	1.25 (1.13 to 1.39)
stage 3	2.70 (2.35 to 3.09)	2.35 (2.07 to 2.68)
stage 4	7.32 (5.78 to 9.29)	7.24 (6.15 to 8.53)
**Grade at diagnosis**		
grade 1- well differentiated	1.00	1.00
grade 2- moderately differentiated	0.97 (0.86 to 1.11)	1.05 (0.95 to 1.15)
grade 3- poorly differentiated	1.05 (0.91 to 1.22)	1.29 (1.16 to 1.45)
grade 4- undifferentiated	0.75 (0.34 to 1.61)	1.14 (0.33 to 3.93)
**Medical history**		
FH of bowel cancer†	0.67 (0.56 to 0.80)	0.88 (0.74 to 1.04)
raised platelets†	1.09 (0.99 to 1.20)	1.20 (1.08 to 1.32)
raised LFTs†	1.34 (1.09 to 1.66)	1.67 (1.46 to 1.90)
statins at diagnosis†	0.84 (0.78 to 0.91)	0.79 (0.75 to 0.84)
aspirin at diagnosis†	0.95 (0.85 to 1.05)	0.92 (0.86 to 0.97)
CVD†	1.36 (1.26 to 1.47)	1.35 (1.26 to 1.44)
type 1 diabetes†	1.63 (0.99 to 2.67)	1.46 (0.84 to 2.54)
type 2 diabetes†	1.20 (1.10 to 1.32)	1.19 (1.11 to 1.28)
**Cancer treatments**		
surgery†	0.65 (0.59 to 0.71)	0.62 (0.56 to 0.67)
Chemotherapy (stage 1) †	1.97 (1.40 to 2.75)	1.71 (1.34 to 2.16)
Chemotherapy (stage 2) †	1.57 (1.36 to 1.81)	1.49 (11.34 to 1.67)
Chemotherapy (stage3) †	1.05 (0.96 to 1.17)	1.08 (0.98 to 1.18)
Chemotherapy (stage4) †	1.38 (1.13 to 1.70)	1.16 (1.00 to 1.34)

Notes $ the Townsend score ranges between -7 (most affluent) and +11 (most deprived). Adjusted hazard ratio is per 5-unit increase. ± in people without chemotherapy
†Adjusted hazard ratio compared with patients without this characteristic. The model for women includes terms of age (2 FP terms 3 3) and body mass index (2 FP terms -2 0). The model for men includes terms of for

age (2 FP terms 3 3) and body mass index (2 FP terms .5 1). There were interactions in men and women between age and stage and an interaction between age and aspirin in women

Supplementary Table 4 Performance of the equations in men and women for (a) all-cause mortality and (b) colorectal cancer mortality in QResearch validation cohort and PHE cancer registry validation cohort at 1 and 10-year follow-up

outcome	*statistic*	*years*	*QResearch cohort*	*PHE cohort*
**women**				
All-cause mortality	D statistic	1	2.027 (1.929 to 2.126)	1.964 (1.933 to 1.995)
	R^2	1	49.52 (47.096 to 51.945)	47.935 (47.148 to 48.722)
	Harrell's C	1	0.814 (0.805 to 0.824)	0.807 (0.804 to 0.809)
	D statistic	10	1.525 (1.453 to 1.597)	1.342 (1.327 to 1.357)
	R^2	10	35.696 (33.523 to 37.87)	30.062 (29.599 to 30.526)
	Harrell's C	10	0.773 (0.766 to 0.78)	0.754 (0.753 to 0.756)
colorectal mortality	D statistic	1	2.096 (1.961 to 2.23)	n/a
	R^2	1	51.173 (47.987 to 54.359)	n/a
	Harrell's C	1	0.823 (0.809 to 0.836)	n/a
	D statistic	10	1.8 (1.702 to 1.898)	n/a
	R^2	10	43.607 (40.936 to 46.278)	n/a
	Harrell's C	10	0.793 (0.78 to 0.806)	n/a
**men**				
All-cause mortality	D statistic	1	2.062 (1.907 to 2.217)	1.939 (1.921 to 1.958)
	R^2	1	50.353 (46.601 to 54.104)	47.309 (46.841 to 47.777)
	Harrell's C	1	0.814 (0.802 to 0.826)	0.795 (0.793 to 0.797)
	D statistic	10	1.465 (1.405 to 1.525)	1.304 (1.293 to 1.315)
	R^2	10	33.878 (32.055 to 35.701)	28.875 (28.525 to 29.224)
	Harrell's C	10	0.766 (0.758 to 0.774)	0.744 (0.742 to 0.745)
colorectal mortality	D statistic	1	2.192 (2.063 to 2.322)	n/a
	R^2	1	53.428 (50.489 to 56.368)	n/a
	Harrell's C	1	0.833 (0.821 to 0.845)	n/a
	D statistic	10	1.798 (1.711 to 1.884)	n/a
	R^2	10	43.548 (41.192 to 45.904)	n/a
	Harrell's C	10	0.793 (0.784 to 0.802)	n/a
